# Group-based microfinance for collective empowerment: a systematic review of health impacts

**DOI:** 10.2471/BLT.15.168252

**Published:** 2016-06-21

**Authors:** Lois Orton, Andy Pennington, Shilpa Nayak, Amanda Sowden, Martin White, Margaret Whitehead

**Affiliations:** a Department of Public Health & Policy, University of Liverpool, Whelan Building, Quadrangle, Brownlow Hill, Liverpool, L69 3GB, England.; b Centre for Reviews and Dissemination, University of York, York, England.; c UKCRC Centre for Diet and Activity Research, University of Cambridge, Cambridge, England.

## Abstract

**Objective:**

To assess the impact on health-related outcomes, of group microfinance schemes based on collective empowerment.

**Methods:**

We searched the databases Social Sciences Citation Index, Embase, MEDLINE, MEDLINE In-Process, PsycINFO, Social Policy & Practice and Conference Proceedings Citation Index for articles published between 1 January 1980 and 29 February 2016. Articles reporting on health impacts associated with group-based microfinance were included in a narrative synthesis.

**Findings:**

We identified one cluster-randomized control trial and 22 quasi-experimental studies. All of the included interventions targeted poor women living in low- or middle-income countries. Some included a health-promotion component. The results of the higher quality studies indicated an association between membership of a microfinance scheme and improvements in the health of women and their children. The observed improvements included reduced maternal and infant mortality, better sexual health and, in some cases, lower levels of interpersonal violence. According to the results of the few studies in which changes in empowerment were measured, membership of the relatively large and well-established microfinance schemes generally led to increased empowerment but this did not necessarily translate into improved health outcomes. Qualitative evidence suggested that increased empowerment may have contributed to observed improvements in contraceptive use and mental well-being and reductions in the risk of violence from an intimate partner.

**Conclusion:**

Membership of the larger, well-established group-based microfinance schemes is associated with improvements in some health outcomes. Future studies need to be designed to cope better with bias and to assess negative as well as positive social and health impacts.

## Résumé

**Objectif:**

Évaluer les conséquences sur les résultats sanitaires de projets de microfinancement de groupe axés sur la responsabilisation collective.

**Méthodes:**

Nous avons recherché dans les bases de données Social Sciences Citation Index, Embase, MEDLINE, MEDLINE In-Process, PsycINFO, Social Policy & Practice et Conference Proceedings Citation Index des articles publiés entre le 1er janvier 1980 et le 29 février 2016. Les articles qui rendaient compte des conséquences sur le plan de la santé liées au microfinancement de groupe ont été inclus dans une synthèse descriptive.

**Résultats:**

Nous avons retenu un essai contrôlé randomisé par groupe et 22 études quasi expérimentales. Toutes les interventions incluses ciblaient des femmes pauvres vivant dans des pays à revenu faible ou intermédiaire. Certaines comprenaient un volet axé sur la promotion de la santé. Les résultats des études de meilleure qualité ont démontré un lien entre l'adhésion à un projet de microfinancement et l'amélioration de la santé des femmes et de leurs enfants. Les améliorations observées se traduisaient par une réduction de la mortalité maternelle et infantile, une meilleure santé sexuelle et, dans certains cas, une diminution des niveaux de violence interpersonnelle. Selon les résultats des rares études dans lesquelles ont été mesurés les changements liés à la responsabilisation, l'adhésion à des projets de microfinancement relativement importants et bien connus a, en règle générale, favorisé la responsabilisation, mais ne s'est pas forcément traduite par de meilleurs résultats sanitaires. Des données qualitatives permettent de penser que le renforcement de la responsabilisation a pu contribuer à des améliorations concernant l'emploi de contraceptifs et le bien-être mental ainsi qu'à la diminution du risque de violence au sein du couple.

**Conclusion:**

L'adhésion à un projet de microfinancement de groupe important et bien connu est associée à l'amélioration de certains résultats sanitaires. De futures études doivent être envisagées pour venir à bout de tout parti pris et évaluer les conséquences positives, mais aussi négatives, sur le plan social et de la santé.

## Resumen

**Objetivo:**

Evaluar el impacto sobre los resultados relacionados con la salud de planes de microfinanciación de grupos basados en el empoderamiento colectivo.

**Métodos:**

Se realizaron búsquedas en las bases de datos de Social Sciences Citation Index, Embase, MEDLINE, MEDLINE In-Process, PsycINFO, Social Policy & Practice y Conference Proceedings Citation Index para encontrar artículos publicados entre el 1 de enero de 1980 y el 29 de febrero de 2016. Artículos que informan sobre los efectos sobre la salud relacionados con la microfinanciación de grupos se incluyeron en una síntesis narrativa.

**Resultados:**

Se identificaron un ensayo controlado aleatorizado y 22 estudios cuasiexperimentales. Todas las intervenciones incluidas apuntaban a mujeres pobres que vivían en países con ingresos bajos o medios. Algunas incluían un componente de fomento de la salud. Los resultados de los estudios de mayor calidad indicaron una relación entre la pertenencia de un plan de microfinanciación y mejoras en la salud de las mujeres y sus hijos. Entre las mejoras observadas se encontraban una reducción de la mortalidad infantil y materna, una mejora de la salud sexual y, en ciertos casos, menores niveles de violencia interpersonal. Según los resultados de los pocos estudios en los que se midieron los cambios en el empoderamiento, la pertenencia de los planes de microfinanciación relativamente grandes y bien constituidos ha dado lugar, en términos generales, a un mayor empoderamiento, pero esto no se traduce necesariamente en unos mejores resultados sanitarios. Las pruebas cualitativas sugirieron que un aumento del empoderamiento puede haber contribuido a mejoras observadas en el uso de métodos anticonceptivos y el bienestar mental, así como un descenso del riesgo de violencia doméstica.

**Conclusión:**

La pertenencia de planes de microfinanciación por grupos mayores y bien constituidos está vinculada a mejoras en algunos resultados sanitarios. Es necesario diseñar futuros estudios para afrontar mejor la parcialidad y para evaluar los impactos sanitarios y sociales positivos y negativos.

## ملخص

**الغرض:**

تقييم التأثير على المحصلات المرتبطة بالصحة، وبرامج التمويل متناهي الصغر للمجموعات القائمة على التمكين الجماعي.

**الطريقة:**

قمنا بالبحث في قواعد البيانات مثل Social Sciences Citation Index، وEmbase، وMEDLINE، وMEDLINE In-Process، وPsycINFO، وSocial Policy & Practice، وConference Proceedings Citation Index للعثور على مقالات منشورة في الفترة ما بين 1 يناير 1980 و29 فبراير 2016. وكانت المقالات التي تبلغ عن التأثيرات الصحية المرتبطة بالتمويل متناهي الصغر القائم على المجموعات مشمولة في أسلوب تجميعي سردي.

**النتائج:**

لقد قمنا بتحديد تجربة واحدة عشوائية عنقودية في بيئة مراقبة و22 دراسة شبه تجريبية. واستهدفت جميع التدخلات المشمولة النساء الفقيرات المقيمات في بلدان منخفضة أو متوسطة الدخل. واشتمل بعضها على أحد مكونات تعزيز الصحة. أشارت النتائج الخاصة بالدراسات عالية الجودة إلى وجود ارتباط بين الانضمام إلى برنامج التمويل متناهي الصغر والتحسينات في مستوى الصحة لدى النساء وأطفالهن. واشتملت التحسينات الملحوظة على تقليل نسب وفيات الأمهات والرضّع، وتحسين الصحة الجنسية، وفي بعض الحالات، خفض مستويات العنف بين الأشخاص. وفقًا للنتائج الصادرة عن بعض الدراسات التي تم فيها قياس مستوى التمكين، عادةً ما أدى الانضمام إلى برامج التمويل متناهي الصغر التي تتميز بأنها كبيرة نسبيًا ومستقرة إلى زيادة مستوى التمكين ولكن هذا الأمر لم يعبر بالضرورة عن تحسين المحصلات الصحية. وأشار الدليل النوعي إلى أن زيادة مستوى التمكين قد تكون ساهمت في التحسينات الملحوظة في استخدام موانع الحمل والسلامة العقلية والحد من خطر التعرض للعنف من شريك الحياة الحميم.

**الاستنتاج:**

إن الانضمام إلى برامج التمويل متناهي الصغر الأكبر والمستقرة والقائمة على المجموعات يرتبط بالتحسينات في بعض المحصلات الصحية. يجب أن تكون الدراسات المستقبلية مصممة للتأقلم بشكلٍ أفضل مع حالات التحيز ولتقييم التأثيرات الاجتماعية والصحية السلبية وكذلك الإيجابية.

## 摘要

**目的:**

旨在评估基于集体授权的群体微型金融计划对健康相关结果的影响。

**方法:**

我们通过社会科学引文索引 (Social Sciences Citation Index)、荷兰医学文摘 (Embase)、MEDLINE、MEDLINE In-Process、PsycINFO、社会政策与实践 (Social Policy & Practice) 以及会议录引文索引 (Conference Proceedings Citation Index) 数据库搜索了发表于 1980 年 1 月 1 日至 2016 年 2 月 29 日之间的文章。关于基于群体的微型金融健康影响的报告文章被纳入在叙述性综述中。

**结果:**

我们鉴定了一项集群随机对照试验和 22 项准实验研究。所有干预措施均针对居住于中低收入国家的贫困妇女。有些研究包含健康改善部分。质量较高的研究结果表明参与微型金融计划与妇女及其子女健康改善之间存在联系。据观察，改善之处包括母婴死亡率的降低、性健康状况更佳，以及在某些情况下，更低水平的人际暴力。根据少数衡量了授权变化的研究结果，参与规模相对较大且体系完善的微型金融计划通常会促进妇女权利获得情况改善，但是这并未绝对性地改善健康结果。定性证据显示妇女权利获得情况的改善可能明显促进了避孕用品的使用、提升了心理健康并且降低了来自亲密伴侣的暴力攻击的风险。

**结论:**

参与规模较大、体系完善的群体微型金融计划与某些健康结果的改善有关。将来研究还需进一步设计，以更好地应对偏见，以及评估负面和正面的社会和健康影响

## Резюме

**Цель:**

Оценить влияние схем группового микрофинансирования на конечные показатели в области здравоохранения на основании расширения коллективных прав и возможностей.

**Методы:**

Авторами был выполнен поиск статей, опубликованных в период между 1 января 1980 года и 29 февраля 2016 года, в базах данных Social Sciences Citation Index (Индекс цитирования по общественным наукам), Embase, MEDLINE, MEDLINE In-Process, PsycINFO, Social Policy & Practice и Conference Proceedings Citation Index (Индекс цитирования по трудам конференций). Статьи, содержащие данные о влиянии группового микрофинансирования на здоровье, были включены в нарративный синтез.

**Результаты:**

Авторы определили одно контролируемое исследование с кластерной рандомизацией и 22 квазиэкспериментальных исследования. Все включенные в анализ мероприятия касались малоимущих женщин, проживающих в странах с низким и средним уровнем дохода. В программу некоторых из них входило укрепление здоровья. В результате исследований высшего качества была установлена связь между участием в схеме микрофинансирования и улучшением здоровья женщин и их детей. Наблюдаемые улучшения включали сокращение смертности среди матерей и новорожденных, укрепление репродуктивного здоровья и в некоторых случаях снижение уровней межличностного насилия. Согласно результатам немногих исследований, в ходе которых измерялись изменения в предоставленных правах и возможностях, участие в сравнительно больших и устойчивых схемах микрофинансирования в целом приводило к увеличению возможностей, но не всегда выражалось в улучшении конечных показателей в области здравоохранения. Качественные данные позволяют сделать предположение, что расширение прав и возможностей, вероятно, поспособствовало наблюдаемым улучшениям в плане применения контрацептивов и психического благополучия, а также снижению риска насилия со стороны партнеров.

**Вывод:**

Участие в сравнительно больших устойчивых схемах группового микрофинансирования обуславливает улучшение в плане некоторых конечных показателей в области здравоохранения. Необходимо разработать дополнительные исследования для уменьшения искажений и для оценки отрицательных и положительных последствий для общества и здравоохранения.

## Introduction

Microfinance initiatives have become popular, particularly in low- and middle-income settings, as a means of promoting rural development,^1^ increasing the bargaining power of women and improving household welfare.^2^ Such has been the enthusiasm for these schemes that, in 2006, the Nobel Peace Prize was awarded jointly to Muhammed Yunus and the Grameen Bank – a microfinance scheme in Bangladesh.

The potential of microfinance to improve health is now being recognized.^3^^–^^5^ The impacts of microfinance initiatives need to be considered in current theory debates about the role that control over destiny plays as a fundamental social determinant of health.^6^ Poor control over destiny, which is a characteristic of women in some societies, can be damaging to population health. In general, population and child health improve and the life expectancies of both men and women increase as the participation of women in decision-making increases.^6^

Group-based microfinance schemes attempt to harness the collective power of mutual support – with members pooling their savings and making small loans to each other so that they can set up small businesses. Most aim to improve the economic power of – and employment opportunities for – women in their immediate community, and many aim to confront engrained discriminatory attitudes to women.^2^ Some aim to facilitate the attendance of girls at school and change attitudes to the paid employment of women outside their homes. The members – who are mostly women – form groups for saving and credit, and are offered literacy classes, legal, social and empowerment training and technical and marketing support (Box 1).

Box 1 **Microfinance schemes based on collective empowerment**

Roughly 5 million poor rural women in Bangladesh are involved in microcredit programmes, most of them associated with the BRAC or Grameen Bank microfinance schemes.

The BRAC scheme is designed for women living in poor and landless households. It involves the formation of women’s groups for saving and credit, training and skills development, functional literacy – including legal and social awareness – and technical and marketing support. Money saved by a group is used to make loans to group members to support income-generation activities such as cottage industries and goat rearing. Sometimes these elements are combined with so-called bolt-on public health components such as the promotion of maternal and child health or family planning.

The main aims of the scheme are to reduce women’s economic dependence on men, strengthen their positions within their families, draw them into the public sphere and expose them to new ideas and education. The theory is that the scheme may influence health in many different ways – e.g. it may increase demand for family planning services and reduce the social costs of fertility regulation, leading to fewer, healthier children and better maternal health. It may also lead to improvements in the care and nutrition of children and so reduce child mortality in general and, particularly, the high rates recorded among girls.

It has been argued that the enthusiasm for microfinance has outstripped the evidence of its effectiveness^7^ and that microfinance schemes have the potential to do harm. Schemes can suffer from so-called mission drift and end up favouring those who are more credit-worthy while excluding the ultra-poor.^8^^–^^12^ In some settings, the imposition of a business model on poor female members of a microfinance scheme may lead to increased debt, repayment stress and exploitation.^13^^,^^14^ The result may be an exacerbation of inequalities rather than a reduction.

We conducted a systematic review of group-based microfinance based on collective empowerment that covered all health conditions and all countries and assessed the impact on health. We addressed three questions: (i) what impact do group-based microfinance schemes based on collective empowerment have on health; (ii) what role does empowerment play in the pathways from microfinance to health impacts; and (iii) do the impacts of the schemes differ based on the ethnicity, sex and/or socioeconomic status of the members?

## Methods

We reviewed evaluations of group-based microfinance in any country, using published systematic review methods,^15^ and assessed the quality of each relevant study using procedures tailored to social interventions in community contexts.^16^

### Search strategy

We searched the databases, Embase, MEDLINE, MEDLINE In-Process, PsycINFO, Social Policy & Practice (Box 2; available at: http://www.who.int/bulletin/volumes/94/9/15-168252), Social Sciences Citation Index and Conference Proceedings Citation Index (Box 3; available at: http://www.who.int/bulletin/volumes/94/9/15-168252) for articles published between 1 January 1980 and 29 February 2016. We checked the reference lists of relevant articles and contacted policy-makers and academics for publications in press and in the grey literature.

Box 2 **Embase, MEDLINE, MEDLINE In-Process, PsycINFO and Social Policy & Practice search strategy**

Titles and abstracts of articles published between 1 January 1980 and 29 February 2016 were searched for the following terms: “micro-credit$”, “microcredit$”, “micro credit$”, “micro-finance$”, “microfinance$”, “micro finance$”, “microsaving$”, “micro-saving$”, “micro saving$”, (Bangladesh and BRAC), (IMAGE adj2 (scheme or intervention or initiative)), Pragati, “Bangladesh Rural Advancement Committee”, “Grameen Bank” and “credit union”.

Box 3 **Social Sciences Citation Index and Conference Proceedings Citation Index search strategy**

Titles and abstracts of articles published between 1 January 1980 and 29 February 2016 were searched for the following terms: “micro-credit$”, “microcredit$”, “micro credit$”, “micro-finance$”, “microfinance$”, “micro finance$”, “microsaving$”, “micro-saving$”, “micro saving$”, IMAGE adj2 (scheme or intervention or initiative)), Pragati, “Bangladesh Rural Advancement Committee”, “Grameen Bank”, “credit union” and (Bangladesh and BRAC).

### Inclusion and exclusion criteria

A report was only included if it described an experimental or quasi-experimental evaluation of a group-based microfinance scheme that: (i) employed collective empowerment strategies; (ii) was targeted at a group with some form of disadvantage; and (iii) was delivered among a free-living population in a community setting. To be included, a report also had to disaggregate data by some measure of socioeconomic status and describe at least one health-related outcome. We also included qualitative reports that related to an included study. No country or language restrictions were applied.

We excluded reports of individual loan schemes that focused solely on poverty alleviation but did not promote group solidarity and empowerment, and reports on schemes that included restrictions on how loans could be used.

### Screening and selection

Titles and abstracts were screened before potentially eligible reports were retrieved in full text and assessed, independently, by two reviewers. Reasons for exclusion were recorded. Disagreements were resolved by discussion or by recourse to a third reviewer.

### Study data

A single reviewer extracted data from each included report and applied a modified version of the quality assessment tool developed by Lorenc et al.^16^ Qualitative studies were assessed using the criteria of Mays and Pope.^17^ A second reviewer checked extractions and appraisals for accuracy and completeness. A narrative synthesis was performed.^18^^,^^19^ Differential impacts were identified – particularly in relation to ethnicity, sex and socioeconomic status. Reporting was based on the PRISMA-Equity 2012 extension guidelines.^20^

## Results

**Fig. 1:**
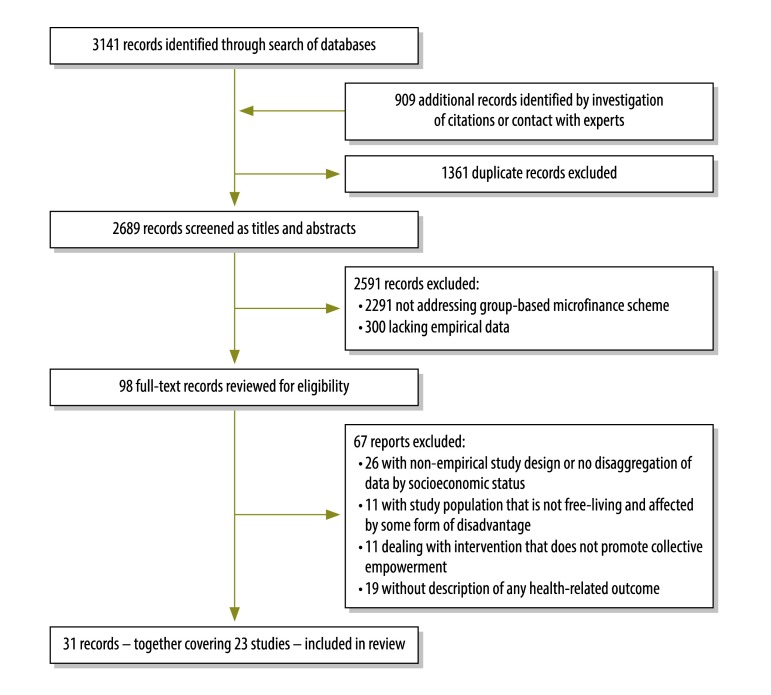
Flowchart showing the selection of studies on group-based microfinance schemes

From 4050 articles, only 31 reports – covering 23 studies (Table 1) – met our inclusion criteria (Fig. 1). The included studies comprised one cluster-randomized controlled trial and 22 quasi-experimental studies that took advantage of naturally occurring comparisons and pre-existing data – e.g. from demographic surveillance systems and health surveys. All of the interventions targeted poor women living in low- or middle-income countries. Most were based in Bangladesh and many focused on women in rural communities. Although we identified some studies of microfinance schemes in central and south America, all but one were excluded because they did not meet the inclusion criteria.

**Table 1:** Summary of the studies included in the systematic review of group-based microfinance schemes

Study no.	Publication	Country and study design	Follow-up period	Intervention and target population	Study participants	Comparison group(s)	Outcome measures	Quality**^a^**
1	Bhuiya and Chowdhury^21^	Bangladesh, controlled before-and-after study	1988–1992 and 1993–1997	BRAC,^b^ poor women	13 549 children of poor women	Children of poor non-members and children of rich non-members	Infant and childhood mortality rates, recorded as survival status on set date for two birth cohorts	Higher
2	Bhuiya et al.^22^	Bangladesh, controlled before-and-after study	1982–1996	BRAC, poor women	Children of poor women from 12 000 households	Children of poor non-members	Childhood mortality rates, recorded as cumulative child survival probability by household	Higher
3	E-Nasreen et al.^23^	Bangladesh, case–control and qualitative case studies	NA	BRAC, poor women	117 neonates born 1999–2000 who died within first 28 days of life	Live children	Neonatal death	Lower
4	Ahmed et al.^24^	Bangladesh, post-intervention study	NA	BRAC, poor women	Poor women from 3817 households	Non-member households that met eligibility for BRAC, and rich non-eligible households	Self-reported illness episodes over last 15 days and health- seeking behaviour	Lower
5	Hamad and Fernald^25^	Peru, post-intervention study	NA	PRISMA,^c^ poor households	1593 adult female members	Long-duration members and short-duration members	Depressive symptoms, contraceptive use, cancer screening: in last year, self-reported days sick in last month	Lower
	Moseson et al.^26^				511 adult female members and 596 of their children aged < 5 years	Long-duration members and short-duration members	Child length-for-age, weight-for-age, anaemia, questions on respiratory infections and diarrhoea in child last 6 months, food security	Lower
	Hamad and Fernald^27^				1593 adult female members	Long-duration members and short-duration members	Age-adjusted BMI, haemoglobin levels and food insecurity	Lower
6	Pronyk et al.^28^	South Africa, cluster-RCT with qualitative component	2 years	IMAGE,^d^ poor women	5156 residents of intervention villages aged 14–35 years	Matched controls from waiting-list villages	Rate of unprotected sex: occurrence at last intercourse with a non-spousal partner in past 12 months, HIV incidence	Highest
	Pronyk et al.^29^				220 female members aged 14–35 years	Matched controls from waiting-list villages	HIV-related communication, access to voluntary counselling and testing, rate of unprotected sex at last intercourse with non-spousal partner	Highest
	Kim et al.^30^				860 women from intervention villages, as 430 matched pairs of members and non-members	Matched controls from waiting-list villages	Physical and sexual violence by spouse or other intimate partner within last year, women’s empowerment	Highest
7	Schuler and Hashemi^31^	Bangladesh, controlled before-and-after study with ethnographic component	Single time-points in 1991 and 1993	BRAC and Grameen Bank,^e^ poor women	1305 poor rural married women aged < 50 years	Eligible non-members and non-eligible non-members	Respondent or partner currently using any form of contraception	Higher
	Schuler et al.^32^				1305 poor rural married women aged < 50 years	Eligible non-members and non-eligible non-members	Relative mobility, economic security, ability to make purchases, freedom from domination and violence, political and legal awareness, participation in political spheres	Higher
8	Souverein et al.^33^	India, longitudinal study	2005–2008	Pragati,^f^ female sex workers	20 330 female sex workers	No comparator – women followed up from first point of contact with scheme until last point of reported contact	STI incidence from syndromic surveillance data, condom use at last paid sex	Higher
9	Amin et al.^34^	Bangladesh, post-intervention study	NA	5 small or medium-sized credit NGOs that adopted loan system of Grameen Bank, NS	3564 rural women, aged < 50 years	Non-loanees from NGO areas and women from non-NGO areas	Current contraceptive use, freedom to manage household expenses, autonomy in movement, authority in family affairs	Lower
	Amin and Li^35^				3564 rural women, aged < 50 years	Non-loanees from NGO areas and women from non-NGO areas	Child immunization, infant and child mortality rate	Lower
10	Desai and Tarozzi^36^	Ethiopia, controlled before-and-after study	2003–2006	Two credit schemes combined with family planning activities, poor women	6440 women aged 15–49 years from poor households	Just the family planning component and just the credit component	Contraceptive use	Lower
11	Schuler et al.^37^	Bangladesh, controlled before-and-after study with ethnographic component	Single time-points in 1991 and 1993	BRAC and Grameen Bank, poor women	1305 poor rural married women aged < 50 years	Eligible non-members and non-eligible non-members	Physical beating by husband in last year, relative mobility, economic security, ability to make purchases, freedom from domination and violence, political and legal awareness, participation in political spheres	Higher
12	Chin^38^	Bangladesh, post-intervention study	NA	BRAC, BRDB and Grameen Bank schemes, NS	1843 rural women	Eligible non-members and non-eligible non-members	Spousal violence directed at women – ever and in last year	Lower
13	Ahmed^39^	Bangladesh, post-intervention study	NA	BRAC, poor women	2044 poor women who were or had been married	Non-member households that met eligibility for BRAC	Violence against women from their husbands in preceding 4 months	Lower
14	Dalal et al.^40^	Bangladesh, post-intervention study	NA	BRAC, BRDB, Grameen Bank, PROSHIKA or any microcredit organization, NS	4465 women aged 15–49 years who were or had been married	Non-members	Moderate physical, severe physical, sexual and any interpersonal violence in last year, economic empowerment	Lower
	Dalal et al.^41^				4925 women aged 15–49 years who were or had been married	Non-members	Last delivery at home without skilled birth attendant or with institutional delivery services, economic empowerment	Lower
15	Bajracharya and Amin^42^	Bangladesh, post-intervention study	NA	BRAC, BRDB, Grameen Bank, PROSHIKA or any microcredit organization, NS	4195 married women aged 15–49 years	Matched non-members	Physical and sexual violence against women by their husbands in last year	Lower
16	Imai and Azam^43^	Bangladesh, household panel survey	1997–1998, 1998–1999, 1999–2000 and 2004–2005	Any microfinance scheme, NS	Women from > 3000 households in 91 intervention villages	Women from neighbouring villages without microfinance	BMI	Higher
17	Khatun et al.^44^	Bangladesh, controlled before-and-after study	3 time-points in 1995–1996	BRAC, poor women	576 children of poor women, aged 6–72 months	Children of poor non-members and children of rich non-members	Stunting, recorded as height-for-age compared with reference median	Higher
18	Jalal and Frongillo^45^	Bangladesh, controlled before-and-after study	3 time-points in 1995–1996	BRAC-based CFPR-TUP initiative, poor women	3551 women and 4131 children from households with child aged 6–60 months	Children and women from non-member households	Nutritional status of women and pre-school children	Higher
19	Deininger and Liu^46^	India, pipeline comparison of current and future members	NA	Indhira Kranthi Patham programme,^g^ poor women	Poor women from 1964 households	People who later joined programme when it came to their village	Energy intake, protein intake and food consumption over last 30 days, social capital, economic empowerment, political empowerment	Higher
20	Doocy et al.^47^	Ethiopia, post-intervention study	NA	WISDOM World Vision Microfinance Institution,^h^ poor households	Clients from 819 rural households and their children aged 6–59 months	Similar incoming clients and community controls	Arm circumference	Lower
21	MkNelly and Dunford^48^	Ghana, repeat cross-sectional study	1993–1996	Credit with Education scheme, poor rural households	308 mother-and-child pairs from poor rural households that had participated in scheme for at least 1 year, with each child aged < 3 years	Non-participants in microfinance areas and waiting-list controls	Child’s weight-for-age and height-for-age plus maternal BMI, self-confidence, vision for the future, status and bargaining power within the household, status and networks in the community	Lower
22	Mohindra et al.^49^	India, post-intervention study	NA	Self-help groups, poor women	928 poor women aged 18–59 years	Women who had been members for > 2 years, women who had been members for < 2 years, non-members living in house with a member and non-members living in a house without a member	Self-assessed physical and mental health, exclusion from health care in last year, whether or not husband is sole decision-maker	Lower
	Mohindra^50^				928 poor women aged 18–59 years	Women who had been members for > 2 years, women who had been members for < 2 years, non-members living in house with a member and non-members living in a house without a member	NA	Higher
23	Ahmed et al.^51^	Bangladesh, post-intervention study	NA	BRAC, poor women	Poor women, from 3 624 households, who were or had been married	Non-member households that met eligibility for BRAC and rich non-eligible households	Three specific questions about emotional stress and its consequences	Lower

BMI: body mass index; BRDB: Bangladesh Rural Development Board; CFPR-TUP: Challenging the Frontiers of Poverty Reduction – Targeting Ultra Poor; HIV: human immunodeficiency virus; IMAGE: Intervention with Microfinance for AIDS and Gender Equity; NA: not applicable; NGO: nongovernmental organization; NS: not specified; RCT: randomized controlled trial; STI: sexually transmitted infection.

^a^ Assessed using procedures tailored to social interventions in community contexts.^16^

^b^ The aims of the BRAC scheme are to improve health and socioeconomic condition through group formation, skill training and collateral-free loans for income-generating activities.

^c^ Scheme based on loan groups run by a nongovernmental organization.

^d^ Includes a large health promotion component related to human immunodeficiency virus.

^e^ The Grameen Bank is a bank for poor rural people that focuses on women.

^f^ Multicomponent microfinance scheme with empowerment approach.

^g^ Largely based on the creation of self-help groups.

^h^ Promotes community banking and solidarity group lending.

Assessment of the included studies revealed that even the higher quality studies were potentially at risk from several forms of selection bias.

### Impacts

#### Mortality and morbidity

Two higher quality longitudinal studies revealed that membership of the BRAC initiative, in Bangladesh, was associated with relatively low infant and child mortality compared with non-membership.^21^^,^^22^ The decline observed in the risk of infant death over a period of 10 years was greatest (53%) for infants of mothers who joined the BRAC scheme, followed by the infants of rich non-members (41%) and then the infants of poor non-members (31%).^21^ The risk of death for the infants of poor BRAC members declined to the level recorded for the infants of rich non-members. There was no association between BRAC membership and survival of children aged 1–5 years. In a further study by the same authors, however, the survival of children aged 1–5 years from poor households was found to be significantly improved if their mothers were BRAC members.^22^

Two lower quality studies found that BRAC membership was associated with lower child mortality^23^ or lower maternal morbidity.^24^ A third study, based in Peru, found no association between length of membership in a group-based microfinance scheme and maternal depression^25^ or child illness.^26^

#### Women’s sexual health

Impacts on sexual health were reported in five evaluations, in Bangladesh, Ethiopia, India and South Africa. Of these evaluations, a study of the highest quality assessed the impacts of scheme membership on women’s sexual health in South Africa.^28^^–^^30^ This was a prospective, matched, cluster-randomized controlled trial – with a strong qualitative component – of the South African Microfinance for AIDS and Gender Equity intervention. This intervention included a large human immunodeficiency virus (HIV) health-promotion element, a microfinance component based on the Grameen Bank model and a so-called Sisters-for-Life gender-focused training component. The microfinance intervention was not associated with any significant changes in rate of unprotected sexual intercourse with a non-spousal partner (relative risk, RR: 1.02; 95% confidence interval, CI: 0.85–1.23) or HIV incidence (RR: 1.06; 95% CI: 0.66–1.69).^28^ Stratifying by age, there was evidence of several beneficial effects on younger participants after two years of involvement with the programme. For example, when compared with women of the same age and poverty from control villages, female participants aged 14–35 years exhibited higher levels of HIV-related communication (adjusted risk ratio, aRR: 1.46; 95% CI: 1.01–2.12), were more likely to have accessed voluntary counselling and testing (aRR: 1.64; 95% CI: 1.06–2.56) and were less likely to have had unprotected sex at last intercourse with a non-spousal partner (aRR: 0.76; 95% CI: 0.60–0.96).^29^ Qualitative data indicated that the intervention had led to a greater acceptance of intra-household communication about HIV and sexuality and increased confidence and skills that, in turn, appeared to have supported the introduction of condoms in sexual relationships.^29^

Findings on the impacts on women’s sexual health assessed in two other higher quality studies were equivocal. In Bangladesh, women in villages participating in the Grameen Bank scheme were more empowered (*P* < 0.01) and more likely to use contraceptives than women in villages without the microfinance scheme (59% versus 43%; *P* < 0.01).^31^ In contrast, no significant association was found between BRAC membership and contraceptive use. Ethnographic data indicated that the Grameen Bank scheme may have increased contraceptive use partly by strengthening women’s economic roles and empowerment and partly by directly promoting family planning and influencing community norms.^32^ Members of microfinance schemes showed relatively high scores for economic security, contribution to family support, freedom and mobility and freedom from domination.^32^ A further longitudinal controlled study in India, evaluated the three-year impact of Pragati – a multicomponent microfinance and health-promotion intervention for female sex workers. In this study, the incidence of sexually transmitted infections decreased and the frequency of condom use at last paid sex increased as microfinance exposure increased over time.^33^

Three lower quality studies also assessed women’s sexual health. One showed associations between microfinance membership in Bangladesh and higher contraceptive use.^34^^,^^35^ The other two, in Ethiopia and Peru, found no association between membership and women’s health.^36^^,^^25^

#### Violence against women

Impacts on interpersonal violence against Bangladeshi or South African women were reported in seven evaluations. The highest quality study found that, after two years, levels of such violence decreased in all four study villages covered by the South African Microfinance for AIDS and Gender Equity intervention but stayed the same or increased in the four control villages.^30^ Women’s membership in the intervention was associated with a reduced risk of exposure to interpersonal violence (aRR: 0.45; 95% CI: 0.23–0.91).^30^ Improvements in all nine of the investigated indicators of women’s empowerment were observed.^30^ Women members had a greater say over household decision-making and felt more able to challenge the acceptability of violence, to expect and receive better treatment from their partners, to leave abusive relationships and to raise public awareness of interpersonal violence in their village.^30^

Another study, also assessed as higher quality, measured violence within spousal relationships in Bangladesh. In this study, women who participated in the BRAC or Grameen Bank schemes – and women who were non-members but lived in Grameen Bank villages –were found to be less likely to be beaten by their husbands than women in control villages.^37^ In this study, the role of empowerment was not clear and the effect of women’s contribution to family support on violence was not significant.^37^ Ethnographic data indicated that, in relatively rich households, women’s membership of a microfinance scheme may have led to an initial increase in violence as the women’s roles and status were redefined and they had increased involvement in the cash economy – leading to a struggle for control over household finances. However, this effect dissipated over time.^37^

Five lower quality studies, all in Bangladesh, gave mixed results. One study found that microfinance participation was associated with a reduction in the likelihood of interpersonal violence against women of 6.8%.^38^ In contrast, after controlling for confounders, two studies found no statistically significant association between microcredit participation and current experience of such violence.^24^^,^^39^ A further lower quality study in Bangladesh found that the better educated women experienced increased exposure to interpersonal violence following membership of a microfinance scheme. This study was poorly adjusted for bias, however.^40^ Another study in Bangladesh, that used propensity score matching to construct an appropriate comparison group of non-members, revealed that levels of interpersonal violence did not differ significantly between members and non-members.^42^

#### Nutrition

Impacts on nutrition were reported in seven evaluations, in Bangladesh, Ethiopia, Ghana, India and Peru. The findings were inconclusive. Some studies showed that scheme membership brought nutritional benefits – mainly for the infants and children of members – and others revealed no significant effects.

Of the three higher quality studies, all from Bangladesh, one found that women from villages with any microfinance scheme showed similar increases in their body mass index to women from neighbouring villages without microfinance.^43^ In another study, the prevalence of stunting was found to be higher (84.6%) among children of poor non-members than among the children of BRAC members (67.3%) or rich non-members (69.4%).^44^ Weight-for-height *z*-scores of children aged 24–35 months from BRAC households were significantly higher (*P* < 0.05) than those of their counterparts from control households.^44^ The final higher quality study found no significant differences between BRAC households and non-member households in terms of three other indicators of nutritional status in children and women.^45^

We included four lower quality studies relating to nutrition in our systematic review: one each from Ethiopia,^47^Ghana,^48^ India^46^ and Peru^26^^,^^27^. Various outcomes were measured, including: women’s and children’s body mass indexes, anthropometry, food security, food consumption and haemoglobin. Findings were mixed, with some schemes showing benefits for microfinance members and others showing no effects. Two studies – one with unadjusted selection bias – found that improvements in several empowerment variables were associated with microfinance membership.^46^^,^^48^

#### Well-being and health-care use

One higher quality study evaluated the Indian Self Help Groups scheme and found that membership was associated with significant reductions in emotional stress and significant increases in the use of health care.^49^ A beneficial spillover effect was also noted for non-participants who lived in a household with a member. No associations were found between participation and self-assessed health or exposure to health risks. This study excluded a socially marginalized group of women – i.e. Paniya women – because they were considered “prone to underestimate their health”. Women members used loans to help cover their health expenditures.^50^

Two lower quality studies in Bangladesh revealed associations between microfinance membership and increases in emotional stress – but only for non-members in households that received loans^51^ – and use of maternal delivery care.^41^ A final study from Peru found that length of participation in a microfinance scheme had no association with women’s access to cancer screening or their number of sick days.^25^

## Discussion

Although we searched for evaluations of group-based microfinance schemes that covered any disadvantaged group in any country, all identified studies that met our inclusion criteria were concerned with the empowerment of poor women in low- or middle-income countries, mainly in Asia.

There is clear evidence of improvements in some important maternal and child health outcomes associated with membership of the long-established BRAC and Grameen Bank microfinance schemes in Bangladesh, including better child survival.^21^^,^^22^ and use of contraceptives.^31^ Results from studies, in a range of countries, on nutritional status and the general health of women who were members of schemes were equivocal. Membership of a microfinance scheme specifically for female sex workers in India was associated with decreases in sexually transmitted infections and increases in condom use during paid sex.^33^ A complex picture emerges for the impact of microfinance on interpersonal violence. The evidence indicates that, while microfinance may eventually lead to a reduction in such violence, an initial increase may occur as gender norms are challenged. The most robust study, a cluster-randomized controlled trial, showed that microfinance schemes can reduce the risk of physical or sexual violence by an intimate partner.^30^

The few included studies that measured aspects of empowerment generally found improvements in empowerment associated with membership of the major schemes^30^^–^^32^^,^^46^^,^^48^ – though these did not necessarily translate into improved health outcomes.^32^ Others have warned that the provision of credit to women does not guarantee their control over the credit’s use and may lead to excess anxiety over the pressure to pay back loans – diminishing, rather than increasing, any sense of empowerment.^30^ The studies with a strong qualitative component provided some of the most convincing evidence of the role of empowerment in the creation of the beneficial effects of microfinance schemes. In the study of the South African Microfinance for AIDS and Gender Equity intervention, participants revealed how reductions in violence resulted from a range of responses – some linked to increasing confidence and empowerment of the women in handling potential flash points.^30^ In Bangladesh, microfinance schemes can empower women by strengthening their economic roles, increasing their say over household decision-making and changing community norms.^32^

We found no relevant studies that assessed the impact of microfinance schemes on ethnic inequalities in health. Some evidence did emerge on how microfinance schemes might help tackle socioeconomic and gender inequalities in health. Most notably, evaluations in Bangladesh indicated that the BRAC microfinance scheme may help to narrow the inequalities in health between boys and girls and the rich and poor.^35^^,^^44^ Such schemes may work not only through improvements in the economic status of the mothers who become members but also through cultural changes in the way girls are valued and nurtured, leading to additional gains for poor girls in relation to poor boys.

The potential for a microfinance scheme to have adverse health impacts was largely unexplored in the evaluations included in our review. Although there have been indications of increased violence between intimate partners as the result of the female empowerment promoted by microfinance, the most robust relevant studies have shown overall reductions in such violence, at least in the long term.^30^^,^^37^ The potential negative health impacts of microfinance schemes as a result of the debt stress associated with the repayment of loans have yet to be investigated in detail.^11^^–^^14^^,^^52^

Research into the positive and negative impacts of microfinance schemes may be particularly challenging, not least because of the potential for selection bias of various forms. Very few of the relevant studies we identified employed the most robust designs. There is a need for more high quality studies that employ appropriate designs that can cope with all of the complexity and potential confounding to be found in the settings in which microfinance schemes must operate. Few, if any, of the evaluations included in our review could disentangle the effects of the main microfinance scheme from those of additional health-promotion and/or health-care components. Even within the microfinance component, it is hard to disentangle the role of the empowerment strategy from that of the poverty-reduction component. Measurement of differential impacts by socioeconomic status is also rare but studies in Bangladesh have shown how this could be done and revealed its potential value.

In conclusion, group-based microfinance schemes represent perhaps the largest experiment in collective empowerment in the world to date. These schemes – and their potential impacts on both health and poverty – deserve close scrutiny. In terms of improvements in selected health outcomes, the evidence coming from the larger, long-established schemes is encouraging. Many questions remain, however, including the scale of the potential for microfinance schemes to do harm. These questions need to be addressed by appropriately designed evaluations that incorporate community-wide assessments of all potential impacts.
